# Monitoring indexes of concrete dam based on correlation and discreteness of multi-point displacements

**DOI:** 10.1371/journal.pone.0200679

**Published:** 2018-07-17

**Authors:** Xiangnan Qin, Chongshi Gu, Erfeng Zhao, Bo Chen, Yanling Yu, Bo Dai

**Affiliations:** 1 State Key Laboratory of Hydrology-Water Resources and Hydraulic Engineering, Hohai University, Nanjing, China; 2 National Engineering Research Center of Water Resources Efficient Utilization and Engineering Safety, Hohai University, Nanjing, China; 3 College of Water Conservancy and Hydropower Engineering, Hohai University, Nanjing, China; 4 Henan Electric Power Survey & Design Institute, Zhengzhou, China; University of Birmingham, UNITED KINGDOM

## Abstract

Monitoring indexes are significant for real-time monitoring of dam performance in ensuring safe and normal operation. Traditional methods for establishing monitoring indexes are mostly focused on single point displacements, and rational monitoring indexes based on multi-point displacements are rare. This study establishes monitoring indexes based on correlation and discreteness of multi-point displacements. The proposed method is applicable when several monitoring points show strong correlation. In this study, principal component analysis (PCA) was introduced for preprocessing the observations of multi-point displacements. Correlation and discreteness of multi-point displacements were extracted and constructed. The correlation and discreteness parts described the integral and local variance of the displacement field. On this basis, the annual maximum values of the correlation and discreteness parts were selected and their probability density functions (PDF) could be generated by employing the principle of maximum entropy. PDF was constructed using maximum entropy method and was least subjective because it barely provided the moment information of the observations. The multi-point monitoring indexes were then determined by the typical low probability method based on the obtained PDFs. Finally, the proposed method was analyzed using a practical engineering and was verified in terms of its feasibility.

## Introduction

Hydraulic structure is an indispensable infrastructure in society because of its comprehensive functions and benefits in flood control, irrigation, and power generation [1, 2]. Safety monitoring indexes based on prototype observations are frequently adopted in hydraulic structures [3–7], and displacement is one of the major monitored items for dam safety [8]. As the monitoring items and data are becoming various and multitudinous, much more attention has been paid to monitoring indexes considering multiple monitoring variables [9–11]. A concrete dam is a dynamic system exposed to influences of various non-deterministic settings, such as environmental variances, hydraulic loads and geological factors. These non-deterministic factors affect the overall displacement of the concrete dam versatilely [12, 13]. Therefore, monitoring indexes based on the interrelationship of multi-point displacements must be developed.

Various methods for establishing monitoring indexes based on multi-point displacements have been proposed in several references [14–16]. Huang et al. [16] investigated the reasonable form of presetting factors of two-dimensional (2D) space mathematical model with multiple survey points, and they established a statistical model for the Danjiangkou Dam. Yu et al. [15] adopted the principal component analysis (PCA) in multivariate analysis of dam monitoring data and they established the overall control region for dam safety monitoring. Yang et al. [14] determined multi-stage warning indicators for the overall deformation of concrete dam considering fuzziness and randomness, and they achieved nondeterministic optimal control.

Monitoring indexes based on multi-point displacements must be determined based on the correlation of the observations. In the present study, PCA [17–20] was introduced to reduce the dimension of observations and obtain the correlation of the observed displacements. PCA is a popular variable reduction technique [8, 9] and converts a p-vector of the observed displacements into a p-vector of principal components. The correlation and discreteness of multi-point displacements were also extracted and established with PCA in this study.

Thereafter, maximum entropy method (MEM) was performed to generate the probability density functions (PDFs) of the correlation and discreteness parts. According to the principle of maximum entropy, MEM selects the PDF with least subjectivity, thereby maximizing the entropy subject to the moment constraints. MEM has been used to achieve the probability distribution in many fields [21–25]. The monitoring indexes of the correlation and discreteness parts could then be decided using the typical low probability method.

The rest of the paper is organized as follows. Section 1 introduces PCA for extracting the principal components of multi-point displacements. The correlation and discreteness parts are also obtained in this section. Section 2 outlines the MEM for generating the PDFs of the annual extreme values of the correlation and discreteness parts. Section 3 demonstrates how the monitoring indexes of the correlation and discreteness parts can be decided using the typical low probability method based on PDFs. Finally, Section 4 analyzes a numerical example for verifying the feasibility of the proposed method.

## PCA for correlation and discreteness of multi-point displacements

False alters, data redundancy and noise effect [15] are the three key problems in dam safety monitoring based on prototype observations. PCA is effective in reducing the frequency of false alarms in consideration of the correlation between numerous observed items, extracting the principal components, realizing data reduction, and reducing the noise effect on the data analysis. Therefore, PCA was introduced in this study in processing the observed multi-point displacements and extracting the correlation and discreteness parts.

### 1.1 Rationale of PCA for multi-point displacements

For *m* monitoring points of dam displacement with certain correlation, *m* simultaneous observations were selected to form the matrix of observed multi-displacement as follows:

$$X=\left[\begin{array}{cccc} x_{1}(t_{1}) & x_{2}(t_{1}) & \cdots & x_{m}(t_{1})\\ x_{1}(t_{2}) & x_{2}(t_{2}) & \cdots & x_{m}(t_{2})\\ \vdots & \vdots & \ddots & \vdots \\ x_{1}(t_{n}) & x_{2}(t_{n}) & \cdots & x_{m}(t_{n}) \end{array}\right]$$ (1)

where *x*_*i*_(*t*_*j*_)(*i*=1,2,⋯,*m*; *j* = 1,2,⋯,*n*) is the displacement at *t*_*j*_ of observation point *i*.

PCA replaces the displacements at *m* monitoring points with *k* principal components, *u*_1_,*u*_2_,⋯,*u*_*k*_(*k* < *m*), which contain the vast majority of the information of the original observations. *k* principal components are the linear combinations of the observed displacements, which can be expressed as follows:

$$\{\begin{array}{c} u_{1}=l_{11}x_{1}+l_{12}x_{2}+\cdots +l_{1m}x_{m}=\sum_{i=1}^{m}l_{1i}x_{i}\\ u_{2}=l_{21}x_{1}+l_{22}x_{2}+\cdots +l_{2m}x_{m}=\sum_{i=1}^{m}l_{2i}x_{i}\\ \vdots \\ u_{k}=l_{k1}x_{1}+l_{k2}x_{2}+\cdots +l_{km}x_{m}=\sum_{i=1}^{m}l_{ki}x_{i} \end{array}$$ (2)

where *l*_*ji*_ are the coefficients of the linear combinations. The principal components must satisfy the following requirements:

1. *u*_*i*_ and *u*_*j*_(*i* ≠ *j*; *i*,*j* = 1,2,⋯,*k*) are independent, i.e. $u_{i}•u_{{}_{j}}^{T}=0$;
2. *u*_*i*_ has the maximum variance among all the linear combinations of *x*_1_,*x*_2_,⋯,*x*_*m*_ which are independent of *u*_1_,*u*_2_,⋯,*u*_*i*−1_.

Based on the aforementioned analysis, the PCA for multi-point displacements determines the linear combinations coefficients *l*_*ji*_, and they are the eigenvectors corresponding to the first *k* largest eigenvalues of the correlation matrix of the multi-point displacements. A two dimensional example was presented as follows to illustrate the mathematical meaning of PCA.

**Fig 1:**
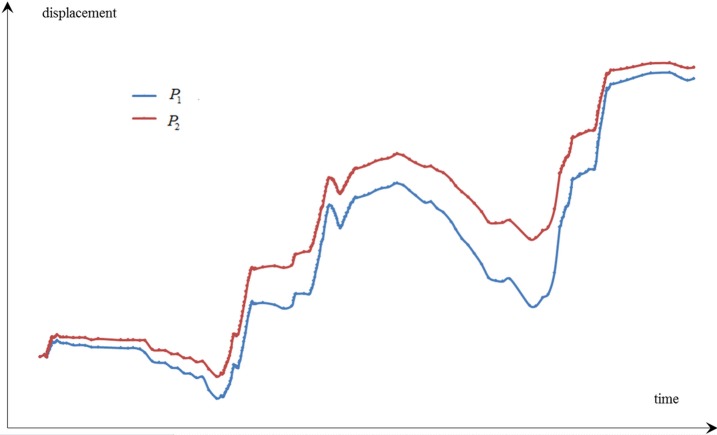
Displacements observations of *P*_1_ and *P*_2_.

**Fig 2:**
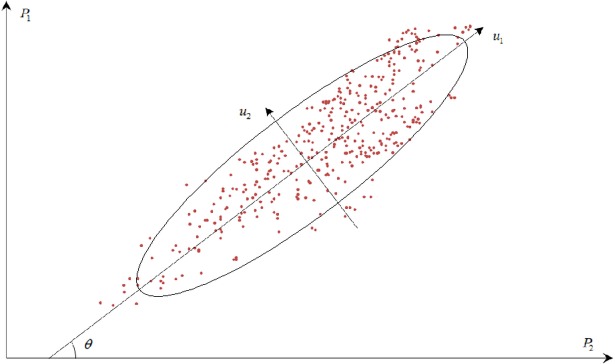
PCA diagram of concrete dam displacements observations in 2D space.

*P*_1_ and *P*_2_ are two observation points with certain correlation. Fig 1 shows the observations of the two points over a certain time period, and Fig 2 illustrates the two principal directions of these two-point observations. Given the correlation between the two observation points, these observations distribute near a straight line and roughly form an ellipse. The coordinate system is then rotated with a certain angle *θ* in 2D space. The major and minor axes of the ellipse are denoted as *u*_1_ and *u*_2_, respectively. The formula of the rotation is presented as follows:

$$\{\begin{array}{c} u_{1j}=x_{1j}\cos\theta +x_{2j}\sin\theta \\ u_{2j}=x_{1j}(-\sin\theta )+x_{2j}\cos\theta \end{array}$$ (3)

where *j* = 1,2,⋯,*N*, and *N* is the times of the observations. The rotation formula can also be expressed in a matrix form as follows.

Y=UX (4)

where *U* is the coordination rotation matrix and $U=\left[\begin{array}{cc} \cos\theta & \sin\theta \\ -\sin\theta & \cos\theta \end{array}\right]$, such that *U*^*T*^ = *U*^−1^,*UU*^*T*^ = *I*.

As shown in Fig 1, the major variance of these observations reflects on *u*_1_ axis after the rotation. If the distribution of the multi-point displacements is expressed in one dimension, then *u*_1_ is the best direction that can guarantee the minimum loss of the original observations. The information of the displacement observations on *u*_1_ is regarded as first principal component, which presents the major correlation of the multi-point displacements. Meanwhile, the information on *u*_2_ is regarded as second principal component. When correlation exists between *m* observation points, the multi-point displacements can be separated in *m* directions in order of importance with orthogonal transformation in multi dimension. The specific steps of PCA for multi-point displacements are presented in the next section.

### 1.2 Steps of PCA for multi-point displacements

The steps of PCA for the correlation and discreteness parts of multi-point displacements are as follows:

**Step 1.** The matrix of multi-point displacement observations was normalized through the following formula:
  $$z_{ij}=\frac{\left(x_{ij}-\bar{x_{j}}\right)}{s_{j}}$$ (5)
  where ${\bar{x}}_{j}=\frac{1}{n}\sum_{i=1}^{n}x_{ij},s_{j}^{2}=\frac{1}{n-1}\sum_{i=1}^{n}(x_{ij}-x_{j}{)}^{2}$. The normalized matrix was then obtained as follows:
  $$Z=\left[\begin{array}{cccc} z_{11} & z_{21} & \cdots & z_{m1}\\ z_{12} & z_{22} & \cdots & z_{m2}\\ \vdots & \vdots & \ddots & \vdots \\ z_{1n} & z_{2n} & \cdots & z_{mn} \end{array}\right]$$ (6)
**Step 2.** The correlation matrix *R* could be generated through the following formula:
  $$R=\frac{1}{n-1}Z^{T}Z$$ (7)
**Step 3.** The secular equation of the correlation matrix *R* was presented as follows:
  $$\left|R-\lambda I_{p}\right|=0$$ (8)
  The solutions of its secular equation were *m* eigenvalues *λ*_1_,*λ*_2_,⋯,*λ*_*m*_(*λ*_1_ ≥ *λ*_2_ ≥ ⋯ ≥ *λ*_*m*_ ≥ 0).
**Step 4.** *m* components of multi-point displacements were calculated through the following formula:
  $$U=ZV^{T}=\left[\begin{array}{cccc} u_{11} & u_{21} & \cdots & u_{m1}\\ u_{12} & u_{22} & \cdots & u_{m2}\\ \vdots & \vdots & \ddots & \vdots \\ u_{1n} & u_{2n} & \cdots & u_{mn} \end{array}\right]=[\begin{array}{cccc} u_{1} & u_{2} & \cdots & u_{m} \end{array}]$$ (9)
  where *V*^*T*^ = [*ν*_1_ *ν*_2_ ⋯ *ν*_*m*_], and *ν*_*i*_ is the real eigenvector corresponding to the eigenvalue *λ*_*i*_.
**Step 5.** The number of the principal components *k* could be determined by calculating the cumulative percent variance (CPV) through the following formula:
  $$CPV(k)=\frac{\sum_{i=1}^{k}\lambda_{i}}{\sum_{i=1}^{n}\lambda_{i}}$$ (10)
  The threshold of CPV, *c*, should be predetermined. When *CPV*(*k*)≥*c*, the first *k* components were the principal components of multi-point displacements. *c* should reach 70%-90% [26].

Based on the aforementioned analysis, *m* principal components of multi-point displacements could be separated into two parts through the following formula:

$$\begin{array}{l} \sum U=u_{1}+u_{2}+\cdots +u_{k}+u_{k+1}+\cdots +u_{m}\\ \;=\sum_{i=1}^{k}u_{i}+\sum_{i=k+1}^{m}u_{i}=U_{p}+U_{e} \end{array}$$ (11)

where *U*_*p*_ includes the vast majority information of the multi-point displacements; this information shows strong correlation and corresponds to the information on *u*_1_ in Fig 2; meanwhile, *U*_*e*_ is the discrete or even uncorrelated information of the multi-point displacements; this information corresponds to the information on *u*_2_ in Fig 2.

### 1.3 Analysis of *U*_*p*_ and *U*_*e*_

The monitoring indexes in this study were determined based on correlation and discreteness of multi-point displacements, corresponding to *U*_*p*_ and *U*_*e*_.

**Fig 3:**
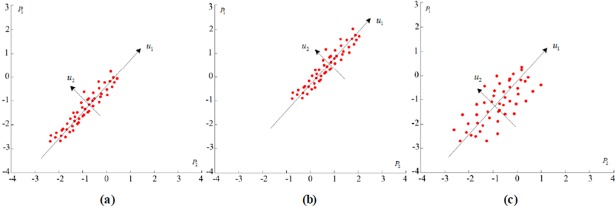
Changing processes related to *U*_*p*_ and *U*_*e*_ in 2D space.

When *U*_*e*_ changes obviously and *U*_*p*_ remains stable, the discreteness of the multi-point displacements increases and the correlation decreases. The variance of the displacement observations on *u*_2_ also increases. This finding corresponds to the changing process from condition (a) to (c) in 2D space in Fig 3. The multi-point displacements show parallel or consistent variation trend if all the included observation points were analyzed as a whole. The sudden drop in correlation of the displacements observations can illustrate that several individual observations locations were interfered by unknown factors. Therefore, the local abnormality of the multiple observations can be detected if all the observation points are guaranteed to be in proper working order.

When *U*_*p*_ changes obviously and *U*_*e*_ remains stable, the correlation and discreteness parts of multi-point displacements remain stable. Meanwhile, the average value of the displacement observations on *u*_1_ changes significantly. This finding corresponds to the changing process from condition (a) to (b) in 2D space in Fig 3. The integral and consistent change of the multi-point displacements can illustrate that the displacement field in this part changes integrally.

Based on the aforementioned analysis, the monitoring indexes of *U*_*p*_ and *U*_*e*_ can be determined for monitoring the integral and local variation of the multi-point displacements according to their PDFs using the typical low probability method. MEM was introduced to approximate the PDFs of *U*_*p*_ and *U*_*e*_ in the next section.

## MEM for the PDFs of correlation and discreteness parts

### 2.1 Maximum entropy principle (MEP)

The Shannon entropy of discrete random variables was defined by the following formula:

$$H(x)=-\sum_{i=1}^{n}p_{i}\ln p_{i}$$ (12)

where *p*_*i*_ is the probability of variable *x*_*i*_(*i* = 1,2,⋯,*n*); $\sum_{i=1}^{n}p_{i}=1,p_{i}\geq 0;\;H(x)$ is the information entropy, which expresses the uncertainty of a stochastic system.

For continuous probability distributions, the Shannon entropy can be defined by the following formula:

$$H(x)=-\int_{R}f(x)\ln f(x)dx$$ (13)

where *f*(*x*) is the PDF of the probability distributions, and $\int_{R}f(x)=1,f(x)\geq 0$.

Formulas (12) and (13) indicate messages in two conditions. On the one hand, the information entropy can be calculated with the two formulas if the probability *p*_*i*_ or the PDF *f*(*x*) is already known; on the other hand, *H*(*x*) can be regarded as the functional of the probability *p*_*i*_ or the PDF *f*(*x*). *H*(*x*) changes with *p*_*i*_ or *f*(*x*) and the probability distribution function can be decided with *H*(*x*). According to the probability distribution statistical inference rules proposed by Jaynes [27], the probability distribution maximizing the information entropy should be selected when based on partial message. Maximum entropy implies that the contrived hypotheses caused by data deficiencies are the minimum and the obtained solution is the most unaffected and objective. MEM is advantageous because of its simple and rapid calculation.

### 2.2 Maximum entropy probability density function

According to the principle of maximum entropy, the probability distribution with minimum deviation maximizes the information entropy *H*(*x*) subject to certain constraints based on the known samples, which is expressed as follows:

$$MAX\;H(x)=-\int_{R}f(x)\ln f(x)dx$$ (14)

subject to

$$\int_{R}f(x)dx=1$$ (15)

$$\int_{R}x^{i}f(x)dx=\mu_{i}\;(i=1,2,\cdots ,N)$$ (16)

where *R* is the domain of integration; *μ*_*i*_(*i* = 1,2,⋯,*N*) is the *i* order origin moment, which can be obtained from the samples.

The first four order origin moments are sufficient in describing the main characteristics of the random variables in many studies [22]. Thus, they were adopted in the present case study. The first origin moment describes the center of the random variables; the second origin moment describes the discreteness of the random variables around the average value; the third origin moment describes the symmetry of the random variables; and the fourth origin moment describes the centralization and decentralization degree of the random variables.

The Lagrange multiplier method was applied to solve this problem and the corresponding Lagrange function was established as follows:

$$L=H(x)+(\lambda_{0}+1)[\int_{R}f(x)dx-1]+\sum_{i=1}^{N}\lambda_{i}[\int_{R}x^{i}f(x)dx-\mu_{i}]$$ (17)

According to stationary value theory (∂*L*/∂*f*(*x*) = 0), Formula (17) can be transformed into the following expression:

$$\frac{\partial L}{\partial f(x)}=-\int_{R}\left[\ln f(x)+1\right]dx+(\lambda_{0}+1)\int_{R}dx+\sum_{i=1}^{N}\lambda_{i}\int_{R}x^{i}dx=0$$ (18)

The analytical form of maximum entropy PDF *f*(*x*) is presented as follows:

$$f(x)=\exp(\lambda_{0}+\sum_{i=1}^{N}\lambda_{i}x^{i})$$ (19)

Formula (19) shows that the solution of maximum entropy PDF can be obtained by determining the Lagrange multipliers (*λ*_0_,*λ*_1_,⋯,*λ*_*N*_).

Substituting Formula (19) into Formula (15) leads to the following formulas:

$$\int_{R}\exp(\lambda_{0}+\sum_{i=1}^{N}\lambda_{i}x^{i})=1$$ (20)

$$\exp(-\lambda_{0})=\int_{R}\exp(\sum_{i=1}^{N}\lambda_{i}x^{i})$$ (21)

$$\lambda_{0}=-\ln\left\{\int_{R}\exp(\sum_{i=1}^{N}\lambda_{i}x^{i})\right\}$$ (22)

Substituting Formulas (19) and (22) into Formula (16) leads to the following formula:

$$\int_{R}x^{i}f(x)dx=\int_{R}x^{i}\exp(\lambda_{0}+\sum_{j=1}^{N}\lambda_{j}x^{j})dx=\frac{\int_{R}x^{i}\exp(\sum_{j=1}^{N}\lambda_{j}x^{j})dx}{\int_{R}\exp(\sum_{j=1}^{N}\lambda_{j}x^{j})dx}=\mu_{i}$$ (23)

For a more convenient numerical calculation, Formula (23) can be transformed into the following expression:

$$1-\frac{\int_{R}x^{i}\exp(\sum_{j=1}^{N}\lambda_{j}x^{j})dx}{\mu_{i}\int_{R}\exp(\sum_{j=1}^{N}\lambda_{j}x^{j})dx}=r_{i}$$ (24)

where *r*_*i*_ are the residuals that can approach zero using a numerical technique. A solution of the Lagrange multipliers (*λ*_0_,*λ*_1_,⋯,*λ*_*N*_) can be generated by non-linear programing to obtain the minimum of the sum of the squared residuals *r*_*i*_.

$$r=\sum_{i=1}^{N}r_{i}^{2}\to \min$$ (25)

Convergence is achieved when *r* < *ε* or |*r*_*i*_| < *ε*, where *ε* is the specified acceptable error. Formula (22) is used to obtain *λ*_0_ with the substitution of the obtained Lagrange multipliers (*λ*_1_,*λ*_2_,⋯,*λ*_*N*_). Eventually, the maximum entropy PDF *f*(*x*) can be determined using Formula (19).

Given that the integration above is difficult to solve by analytical methods, a numerical integration is needed. The domain of integration *R* should be preprocessed and the upper and lower bounds of the PDFs should be assumed in advance. Considering that the PDF *f*(*x*) of random variables *x* is generally characterized by its thin tails, the integral value of *f*(*x*) in the infinite domain (−∞,+∞) can be approximated by that in the finite domain (*a*,*b*). The residual error should be reduced to be acceptable if the finite domain (*a*,*b*) is sufficiently wide. For example, the residual error is 0.27% if (*a*,*b*) was set as (*μ* − 3*σ*,*μ* + 3*σ*) for random variables *x* obeying normal distribution. (*a*,*b*) was set as (*μ* − 5*σ*,*μ* + 5*σ*) conservatively in the following numerical example considering that the practical probability distribution may be skewed.

The PDFs of the annual maximum and minimum values of *U*_*p*_ and *U*_*e*_ can be approximated using MEM. The monitoring indexes are determined in the next section.

## Monitoring indexes of correlation and discreteness

Based on the PDFs of the annual maximum and minimum values of *U*_*p*_ and *U*_*e*_, the monitoring indexes $\left(\delta_{\min}^{p},\delta_{\max}^{p}\right)$ and $\left(\delta_{\min}^{e},\delta_{\max}^{e}\right)$ can be determined given certain significance level *α* [28, 29]. When *U*_*p*_, the correlation part of observed multi-point displacements, exceeds $\left(\delta_{\min}^{p},\delta_{\max}^{p}\right)$, the integral displacement of the dam is in a warning situation. Therefore, forewarning measures must be adopted against probable danger. When *U*_*e*_, the discreteness part of observed multi-point displacements, exceeds $\left(\delta_{\min}^{e},\delta_{\max}^{e}\right)$, local abnormality may occur in the observed region. Thus, timely inspection must be adopted to guarantee the safety and forewarning measures must be taken.

The corresponding exceedance probability and monitoring indexes $\left(\delta_{\min}^{p},\delta_{\max}^{p}\right)$ and $\left(\delta_{\min}^{e},\delta_{\max}^{e}\right)$ can be derived with the following formula:

$$\{\begin{array}{c} P(\delta >\delta_{\max})=\alpha =\int_{\delta_{\max}}^{\infty }f_{\max}(\delta )d\delta \\ P(\delta <\delta_{\min})=\alpha =\int_{-\infty }^{\delta_{\min}}f_{\min}(\delta )d\delta \end{array}$$ (26)

where *f*_min_(*δ*) and *f*_max_(*δ*) are the PDFs of the annual maximum and minimum values of *U*_*p*_ and *U*_*e*_.

**Fig 4:**
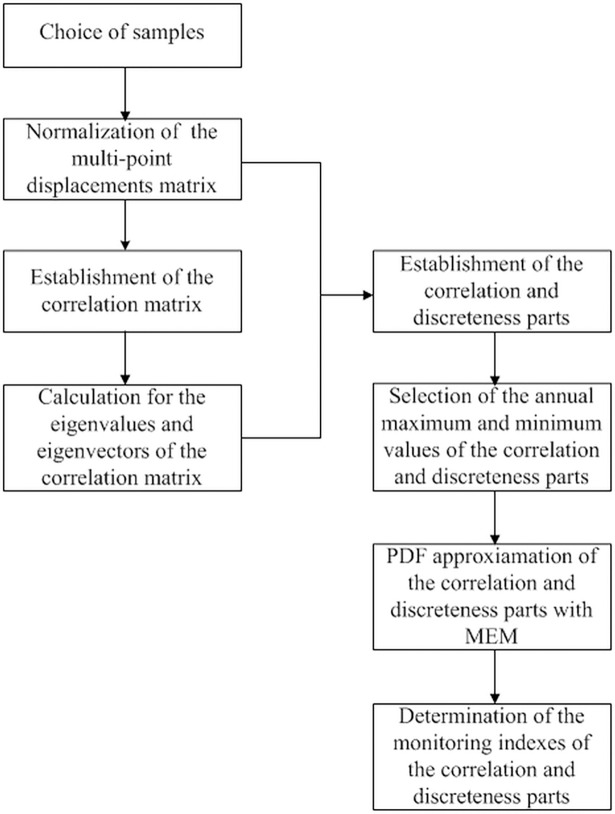
Flowchart for the determination of monitoring indexes of the correlation and discreteness parts.

The flowchart for the determination of the multi-point displacement monitoring indexes of the correlation and discreteness parts is presented in Fig 4.

## Numerical example

**Fig 5:**
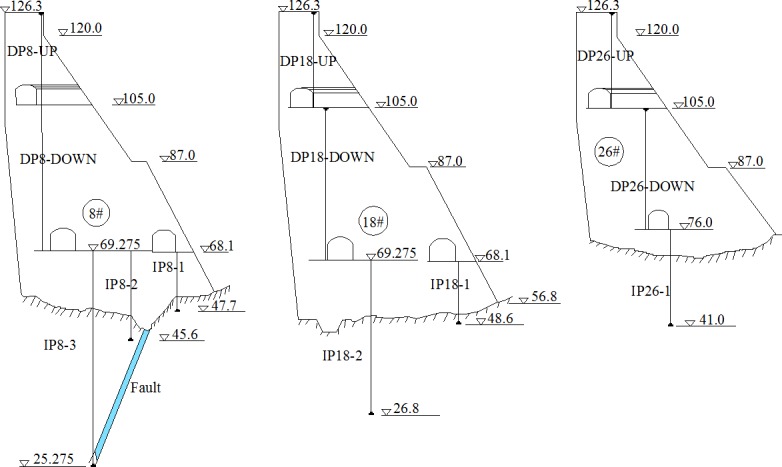
Layout of the direct pendulums in 8#, 18#, 26# dam sections.

**Fig 6:**
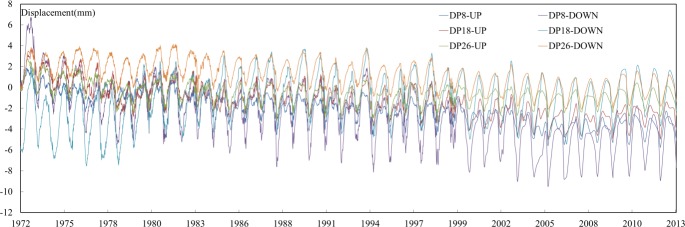
Process lines of the multi-point radial displacements observations.

The concrete gravity arch dam with variable radii located in the upstream of Qingyi River, a tributary of Yangzi River, is an important part of a comprehensive middle-sized hydropower project. The dam was constructed from August 1958 and was completed in 12 years. The elevation of the dam crest is 126.3 m, the maximum height of dam blocks is 76.3 m, and the dam consists of 28 blocks. The dead water level of the reservoir is 101.0 m and the normal high water level is 119.0 m. The total capacity of the dam is 2.825×10^8^ m^3^. The vertical observation includes the direct and indirect pendulums, which are mainly buried in dam galleries or dam abutments. The direct pendulums (DP8-UP, DP8-DOWN, DP18-UP, DP18-DOWN, DP26-UP, DP26-DOWN) in 8#, 18#, 26# dam sections (Fig 5), were selected for determining the monitoring indexes. The observations from July 18, 1972 to July 11, 2013 were analyzed. The observations of the multi-point radial displacements and water level are shown in Fig 6. The radial horizontal displacement is positive when it moving towards downstream and negative when it moving towards the upstream.

In this study, the two-stage monitoring indexes of correlation and discreteness were determined according to the practical operation of the project. The significance level *α* was assumed as 0.05 for the primary monitoring indexes and 0.01 for the secondary monitoring indexes. The reliability coefficients of the monitoring indexes were 95% and 99%, respectively.

### 4.1 Extraction of correlation and discreteness parts using PCA

**Fig 7:**
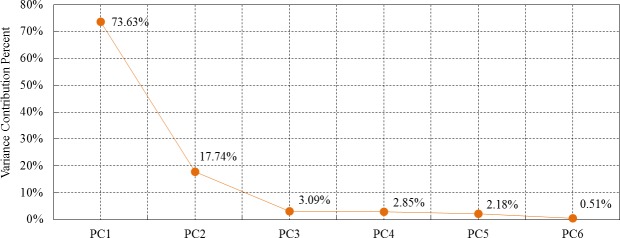
Variance contribution percent of the principal components.

**Fig 8:**
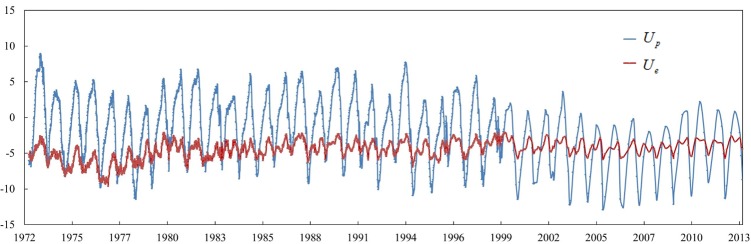
Process lines of correlation and discreteness parts.

PCA was conducted with the observations of the six selected observation points, and the variance contribution percent of the six extracted components are shown in Fig 7. The eigenvalues, variance contribution percent and CPVs are listed in Table 1. The threshold of CPV, *c*, was set as 90% in this study. The information included in the first two components reached 91.37%, exceeding 90%. Therefore, the correlation part *U*_*p*_ is the sum of the first two components and the discreteness part *U*_*e*_ is the sum of the last four components, which were expressed as follows:

$$\begin{array}{l} U_{p}=u_{1}+u_{2}=\sum_{i=1}^{2}u_{i}\\ U_{e}=u_{3}+u_{4}+u_{5}+u_{6}=\sum_{i=3}^{6}u_{i} \end{array}$$ (27)

where *u*_*i*_ are the extracted principal components. The process lines of *U*_*p*_ and *U*_*e*_ are shown in Fig 8.

**Table 1:** Eigenvalues, variance contribution percent and CPVs of the components.

Principal Components	PC1	PC2	PC3	PC4	PC5	PC6
Eigenvalue	1529.94	368.69	64.29	59.19	45.37	10.52
Variance Contribution Percent	73.63%	17.74%	3.09%	2.85%	2.18%	0.51%
CPV	73.63%	91.37%	94.46%	97.31%	99.49%	100.00%

### 4.2 Generation of PDFs of *U*_*p*_ and *U*_*e*_ using MEM

In determining the monitoring indexes $\left(\delta_{\min}^{p},\delta_{\max}^{p}\right)$ and $\left(\delta_{\min}^{e},\delta_{\max}^{e}\right)$, the annual extreme values of *U*_*p*_ and *U*_*e*_ from 1972 to 2012 were selected to generate PDFs. The first four moments for the annual extreme values of *U*_*p*_ and *U*_*e*_ are listed in Table 2.

**Table 2:** First four moments for the annual extreme values of *U*_*p*_ and *U*_*e*_.

Moments	*U*_*p*_		*U*_*e*_	
	Annual maximum values	Annual minimum values	Annual maximum values	Annual minimum values
0	1.00	1.00	1.00	1.00
1st	3.58	-9.13	-3.00	-6.14
2nd	19.92	87.71	9.43	38.97
3rd	117.04	-881.22	-31.23	-256.61
4th	752.70	9193.06	109.13	1760.01

The first four moments for the annual extreme values of *U*_*p*_ and *U*_*e*_ were substituted into Formula (24), and the non-linear least square method was performed to obtain the solution of the five Lagrange multipliers (*λ*_1_,*λ*_2_,*λ*_3_,*λ*_4_). *λ*_0_ can then be solved by substituting Lagrange multipliers (*λ*_1_,*λ*_2_,*λ*_3_,*λ*_4_) into Formula (22). Table 3 presents the solutions for these Lagrange multipliers.

**Table 3:** Lagrange multipliers of the PDFs for *U*_*p*_ and *U*_*e*_.

Lagrange multipliers	*U*_*p*_		*U*_*e*_	
	Annual maximum values	Annual minimum values	Annual maximum values	Annual minimum values
*λ*_0_	-2.6149	-53.6424	-74.4431	-29.4397
*λ*_1_	0.2725	-22.7234	-37.6518	-28.0643
*λ*_2_	-0.0947	-3.7109	-6.9236	-9.4202
*λ*_3_	0.0245	-0.2675	-0.5426	-1.2896
*λ*_4_	-0.0023	-0.0072	-0.0157	-0.0648

Accordingly, the corresponding maximum entropy PDFs for the annual extreme values of *U*_*p*_ and *U*_*e*_ can be expressed as follows:

$$f_{\max}(U_{p})=\exp(-2.6149+0.2725x-0.0947x^{2}+0.0245x^{3}-0.0023x^{4})$$ (28)

$$f_{\min}(U_{p})=\exp(-53.6424-22.7234x-3.7109x^{2}-0.2675x^{3}-0.0072x^{4})$$ (29)

$$f_{\max}(U_{e})=\exp(-74.4431-37.6518x-6.9236x^{2}-0.5426x^{3}-0.0157x^{4})$$ (30)

$$f_{\min}(U_{e})=\exp(-29.4397-28.0643x-9.4202x^{2}-1.2896x^{3}-0.0648x^{4})$$ (31)

### 4.3 Determination of the monitoring indexes of *U*_*p*_ and *U*_*e*_

In the case of the correlation part *U*_*p*_, if *α* = 5%, then the primary warning monitoring index is (-12.52,7.52); if *α* = 1%, then the secondary warning monitoring index is (-13.37,8.51). In the case of the discreteness part *U*_*e*_, if *α* = 5%, then the primary warning monitoring index is (-8.30,-2.07); if *α* = 1%, then the secondary warning monitoring index is (-9.99,-1.77).

**Fig 9:**
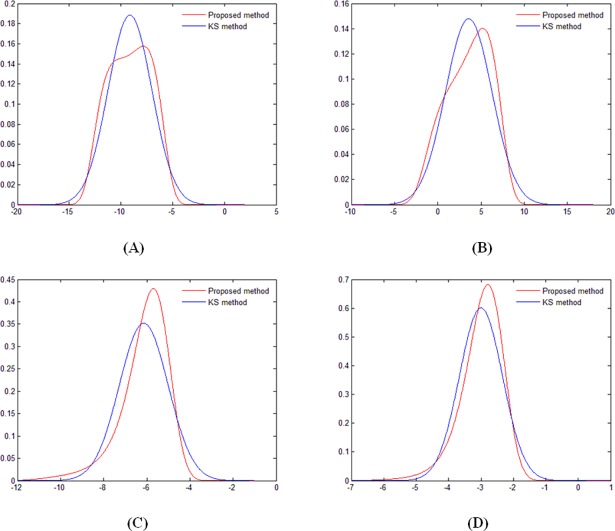
**Probability density curves approximated using proposed method and the KS method:** (A) Annual minimum values of *U*_*p*_; (B) Annual maximum values of *U*_*p*_; (C) Annual minimum values of *U*_*e*_; (D) Annual maximum values of *U*_*e*_.

Table 4 presents the two-stage monitoring indexes of *U*_*p*_ and *U*_*e*_ decided using the proposed method and the KS method. The probability density curves of the two methods are shown in Fig 9. The results obtained by the proposed method were close to those by the KS method, and the probability density curves of these two methods were relatively consistent. However, the probability distributions approximated by the KS method were derived from the reference distributions and had high subjectivity. MEM generated the probability distributions merely considering the moment information of the multi-point displacement observations and was thus more objective. Therefore, the monitoring indexes of the proposed method were more rational and recommended to be used for the practical monitoring and operation.

**Table 4:** Two-stage monitoring indexes of *U*_*p*_ and *U*_*e*_.

Part of the multi-point displacements	Method	Confidence level	
		The primary warning monitoring indexes	The secondary warning monitoring indexes
*U*_*p*_	Proposed method	(-12.52,7.52)	(-13.37,8.51)
	KS method	(-12.61,8.02)	(-14.05,9.90)
*U*_*e*_	Proposed method	(-8.30,-2.07)	(-9.99,-1.77)
	KS method	(-8.01,-1.91)	(-8.78,-1.46)

## Conclusions

This paper presents a method for establishing monitoring indexes of correlation and discreteness of multi-point displacements for concrete dam using PCA and MEM. The correlation and discreteness parts of multi-point displacements were extracted and constructed using PCA, which can describe the integral and local variation trend of the dam displacement. The PDFs of the two parts were approximated by MEM, which is an effective approach to establish a probability density distribution with least subjectivity given a finite number of moments. The monitoring indexes of the two parts could be determined given a certain significance level. The feasibility of the proposed method was demonstrated by a numerical example. The numerical results show that the proposed method could determine rational and accurate multi-point monitoring indexes for concrete dam displacement. A comparison of the results from the proposed method and the KS method confirms the accuracy of the proposed method. The monitoring indexes determined by the proposed method were recommended because of their least subjectivity. However, the accuracy of the monitoring indexes obtained by the proposed method depends on the observation data under most unfavorable load combination, and a structural mechanic analysis is ignored in this study. Further research will be directed to improve the insufficiency and make the monitoring indexes highly rational.

## Supporting information

S1 File (XLSX)
